# Professor Jones’ Medical and Surgical Memoirs

**Published:** 1887-05

**Authors:** 


### PROFESSOR JONES' MEDICAL AND SURGICAL MEMOIRS.

This book, an advertisement of which may be found on our twelfth advertising page, is a very able work. It must have required a prodigious amount of labor and expense. Professor Jones will be remembered as the able and indefatigable Surgeon in the Provisional Army of the Confederate States—a man of profound learning and of untiring energy in medical and scientific investigation. We have not space, and feel that, if we had, we could not do justice to Professor Jones or his able work. It is a work which contains an immense amount of interesting information, and on subjects but little understood. Our readers should by all means procure a copy of this book, which they may do by addressing Prof. Joseph Jones, at New Orleans. See advertisement.

